# Contemporary Theory and Research

**Published:** 1893-05-06

**Authors:** 


### CONTEMPORARY THEORY AND RESEARCH.

### TRANSPLANTATION OF FROG'S SKIN.

A CURIOUS operation was reported before the Société d'Ophtalmologie in February. A boy of thirteen, after an injury to his eyelid, had it so severely contracted that he could no longer close his eye. Accordingly an incision was made in the eyelid by M. Gillet de Grandmont, and tiny fragments of frog's skin were inserted in a kind of chequer work. It adhered perfectly, and the wound was completely healed over. After about five months the eyelid recovered its power of movement. A tiny transverse line across the lid is the only sign visible of the fragments borrowed from the frog.

### CARRIER FALCONS.

M. SMOÏLOFF, a lieutenant in the Russian army, has contributed to the *Revue des Sciences* the result of some experiments in training falcons to carry despatches. Compared with pigeons, falcons are in more than one respect superior as carriers. A pigeon travels from eight to ten leagues an hour, while a falcon, it appears, can cover fifteen leagues in a hour and keeps up this rate of speed for fifteen hours at a time. The falcon can also of course be entrusted with a heavier budget; he is less easily affected by atmospheric changes, encounters fewer dangers on the way, and can rarely become the victim of an enemy stronger than himself.

### THE DIPLOPOMETER.

IN order to render the definition possible of the degree of error in double images, M. Galezowski has constructed an apparatus composed as follows: A stereoscope with two lenses, in front of which glasses may be placed; an unpolished glass cut in squares placed a little behind, with every vertical and horizontal line numbered; a vertical rod placed at a distance of about a yard, at the end of which is a square lamp supporting another little lamp, which preserves an upright position in all movements, and which shines on to a square model turning on its centre and describing a circumference parallel to the stereoscope. The patient can see two reflections of the lamp on the unpolished glass—one blue, the other red. Each movement of the lamp can be defined by the divisions marked on the chequered glass.

### HICCOUGHS.

HICCOUGHS become sometimes a source of danger by their incontrollability, and are always difficult to cure. In the *Journal d'Hygiène* is a description of the method adopted by Dr. Leloir for the alleviation of this distressing affection. He found that by compressing the left phrenic nerves the hiccoughs disappeared almost instantly. In one instance a little girl was thus cured who had suffered for a year from uncontrollable hiccoughs, recurring every half minute, anti-spasmodics having proved entirely useless. M. Leloir forcibly compressed the left phrenic nerve between the two sterno clavicular bands of the sterno-cleido-mastœdian muscle. The compression of the fingers, painful enough, lasted three minutes, at the end of which the hiccoughs had completely disappeared, and never afterwards recurred. This method has since been frequently applied, and forms an interesting explanation of the researches of Vulpian, Charcot, and Brown-Sequard on the therapeutic effect of the excitement of the peripheric nerves.

### TOLYSAL.

TOLYSAL, discovered by Dr. Henning, of Kœnigsberg, is recommended by him as an anti-pyretic and remedy for rheumatism. It is derived from another body known as tolypyrine. It consists of little crystals, almost colourless, bitter to the taste; the point of fusion is between 101 deg. and 102 deg.; it dissolves with difficulty in ether or water, but is easily soluble in alcohol or acetic ether. It is given in doses of from three to six grammes every half-hour in acute rheumatism, and has succeeded invariably without ill-effects. In smaller doses, continued for several days, it has also proved very beneficial in cases of chronic rheumatism. The lowering of the temperature takes place an hour after the administration of the first dose, and lasts a good time. The pulse and respiration are lowered at the same time, but never to the extent of collapse. Tolysal also procures sleep to the patient. It may be employed in cases where other medicaments, such as salicylate of soda, antipyrin, phenacetine, &c., have failed. Like all bodies derived from phenol, it has antiseptic and antifermentescible properties. Being with difficulty soluble in water, tolysal is more easily administered in cachets or tablets.

